# Evaluation of implementation and effectiveness of digital adherence technology with differentiated care to support tuberculosis treatment adherence and improve treatment outcomes in Ethiopia: a study protocol for a cluster randomised trial

**DOI:** 10.1186/s12879-021-06833-x

**Published:** 2021-11-10

**Authors:** Amare W. Tadesse, Zemedu Mohammed, Nicola Foster, Matthew Quaife, Christopher Finn McQuaid, Jens Levy, Kristian van Kalmthout, Job van Rest, Degu Jerene, Tofik Abdurhman, Hiwot Yazew, Demekech G. Umeta, Demelash Assefa, Gedion T. Weldemichael, Ahmed Bedru, Taye Letta, Katherine L. Fielding

**Affiliations:** 1 TB Centre, Department of Infectious Disease Epidemiology, London School of Hygiene & Tropical Medicine (LSHTM), London, UK; 2 KNCV Tuberculosis Foundation, Addis Ababa, Ethiopia; 3 TB Modelling Group, TB Centre, and Centre for Mathematical Modelling of Infectious Diseases, Department of Infectious Disease Epidemiology, London School of Hygiene & Tropical Medicine (LSHTM), London, UK; 4 KNCV Tuberculosis Foundation, The Hague, the Netherlands; 5 National Tuberculosis Control Program, Ethiopian Ministry of Health, Addis Ababa, Ethiopia; 6 School of Public Health, University of the Witwatersrand, Johannesburg, South Africa

**Keywords:** Tuberculosis, Smart pillbox, Medication label, Trial, Cluster randomised, Pragmatic, Ethiopia

## Abstract

**Background:**

Digital adherence technologies (DATs) are recommended to support patient-centred, differentiated care to improve tuberculosis (TB) treatment outcomes, but evidence that such technologies improve adherence is limited. We aim to implement and evaluate the effectiveness of smart pillboxes and medication labels linked to an adherence data platform, to create a differentiated care response to patient adherence and improve TB care among adult pulmonary TB participants. Our study is part of the Adherence Support Coalition to End TB (ASCENT) project in Ethiopia.

**Methods/Design:**

We will conduct a pragmatic three-arm cluster-randomised trial with 78 health facilities in two regions in Ethiopia. Facilities are randomised (1:1:1) to either of the two intervention arms or standard of care. Adults aged ≥ 18 years with drug-sensitive (DS) pulmonary TB are enrolled over 12 months and followed-up for 12 months after treatment initiation. Participants in facilities randomised to either of the two intervention arms are offered a DAT linked to the web-based ASCENT adherence platform for daily adherence monitoring and differentiated response to patient adherence for those who have missed doses. Participants at standard of care facilities receive routine care. For those that had bacteriologically confirmed TB at treatment initiation and can produce sputum without induction, sputum culture will be performed approximately 6 months after the end of treatment to measure disease recurrence. The primary endpoint is a composite unfavourable outcome measured over 12 months from TB treatment initiation defined as either poor end of treatment outcome (lost to follow-up, death, or treatment failure) or treatment recurrence measured 6 months after the scheduled end of treatment. This study will also evaluate the effectiveness, feasibility, and cost-effectiveness of DAT systems for DS-TB patients.

**Discussion:**

This trial will evaluate the impact and contextual factors of medication label and smart pillbox with a differentiated response to patient care, among adult pulmonary DS-TB participants in Ethiopia. If successful, this evaluation will generate valuable evidence via a shared evaluation framework for optimal use and scale-up.

*Trial registration*: Pan African Clinical Trials Registry PACTR202008776694999, https://pactr.samrc.ac.za/TrialDisplay.aspx?TrialID=12241, registered on August 11, 2020.

**Supplementary Information:**

The online version contains supplementary material available at 10.1186/s12879-021-06833-x.

## Background

Globally, an estimated 10.0 million people became ill with tuberculosis (TB) in 2019, with 7.1 million diagnosed, treatment success rate of 85%, and about 1.4 million deaths [1]. TB treatment is the core of TB control and prevention, but poor adherence to treatment can lead to disease relapse, drug-resistance, and forward TB transmission [2]. Poor treatment outcomes are disproportionately high in low-income countries and among low-socio-economic groups within countries [1]. The World Health Organization (WHO) End TB Strategy, in alignment with the Sustainable Development Goals (SDG), has set ambitious goals to end the global TB epidemic by 2035 [3]. This strategy requires high-impact interventions to ensure equitable access to high-quality diagnosis, treatment, care, and prevention, without imposing catastrophic costs, to people with TB.

The WHO-recommended Directly Observed Treatment (DOT) approach has been the most widely implemented strategy to support administration of TB treatment and ensure that patients successfully complete the treatment period. While significant improvements in TB treatment outcomes have been observed after the introduction of DOT, global treatment success rates for both drug-sensitive (DS)-TB and particularly drug-resistant (DR)-TB remain below the 90% target for 2025 set out in WHO’s End TB strategy [1, 3]. The implementation of facility-based DOT places a significant burden on both patient and health systems [4–7]. For patients, DOT is associated with stigma, loss of autonomy and lost income. For providers DOTS is resource intensive and a burden on busy health care facilities. Furthermore, the traditional DOT approach assumes that all TB patients require the same level of monitoring and support, rather than focusing care on patients that are at higher risk for non-adherence and poor outcomes, and therefore in need of intensified support [8].

Adherence to TB treatment is essential to attain durable cure and avoid the emergence of drug resistance [9]. It is also an important element of clinical trials of TB treatment as it strongly influences the treatment outcome and therefore evaluation of the intervention [10]. While there are variations across studies in classifying adherence, definitions of adherence to TB treatment have emphasized patients’ roles in medication-taking throughout the treatment course following the recommendations from the provider [2, 11]. Adherence to treatment has been shown to be influenced by patient characteristics, treatment regimen complexity, and the health care setting [2, 11], and the use of adherence interventions such as electronic medication monitors and mobile text messaging has shown to improve TB treatment outcomes [12–15]. Adherence technologies may be expected to vary in utility by patient characteristics as well as cultural and health care setting.

Currently, a number of digital adherence technologies (DATs), such as electronic medication monitors and text messaging, are available, offering approaches to improve treatment adherence [16]. These technologies are digital tools that utilize mobile phone, computer, web-based and/or electronic sensor technology to support patient care and adherence. These technologies often employ reminders to patients for taking treatment, as well as the capture of detailed, timely, patient-specific adherence data to be made available to the patient’s health-care provider for monitoring. Electronic medication boxes are medication monitoring devices (often with audio-visual reminders) that store TB medications and which record and transmit patient box access to give the provider the patients’ dosing history. The medication sleeve is also a type of electronic medication monitor that consists of medication blisters wrapped in special envelopes with printed codes. Patients use these codes when making a toll-free call/text to let their health care provider know when they have taken their medication [16]. Such digital innovations are beneficial because they may remind patients to take their medications. Digital adherence technologies may be employed with a web-based platform to compile dosing histories that allow health-care providers to review adherence daily. Although the End TB Strategy recommends using digital adherence technologies, evidence that such technologies improve adherence and treatment outcomes is still limited [17].

Recent randomised studies in countries in Africa and Asia documented mixed results regarding effectiveness of medication monitoring to reduce poor medication adherence and treatment outcomes [18–24]. However, it is not clear whether these findings can be generalised to real-life circumstances that are not reflected in randomised controlled trials. Furthermore, the patient and health-care provider acceptability and uptake of these tools have been shown to be vary across countries and settings [25–27]. Cultural or material circumstances may operate differently on the utility or acceptability of DATs to deliver the targeted treatment support that is needed to meet the End TB goals. Data from individual trials might not typically provide policy makers with all the information needed to replicate their success in real-life settings and specific contexts.

Ethiopia is among the 30 high TB and TB/ HIV burden countries, with annual estimated TB incidence of 157/100,000 populations and death rate of 19 per 100,000 populations for 2019 [1]. Since 1997, Ethiopia has adopted Directly observed treatment, short-course (DOTS) as its core treatment strategy, with the development of the first combined Tuberculosis and Leprosy Prevention and Control Program manual [28] and the national control program endorsed the global STOP TB and END TB strategies over subsequent years [28, 29]. However, non-adherence to TB treatment and poor treatment outcomes remain the major barriers to achieve the national END TB Strategy targets [30–32]. As TB remains a public health problem with significant burden among affected and at-risk populations in Ethiopia, the country’s national strategic plan recommends evidence-based and high-impact interventions that will keep the nation on track to end the TB epidemic [29]. The implementation of DATs has the potential to promote a cost-effective, patient-centred approach to improve TB treatment adherence across a range of settings and thereby reduce TB incidence and mortality to end TB as a public health problem. The Adherence Support Coalition to End TB (ASCENT) Consortium members [Koninklijke Nederlandse Centrale Vereniging (KNCV) Tuberculosis Foundation, The Aurum Institute, London School of Hygiene & Tropical Medicine, and Program for Appropriate Technology in Health (PATH)] in partnership with the governments of five countries (Ethiopia, Philippines, South Africa, Tanzania, and Ukraine) are implementing the ASCENT project, that will contribute evidence to support the adoption and uptake of DATs. In Ethiopia, this evidence will be generated through a pragmatic cluster randomized trial to implement and assess the effectiveness of medication labels and smart pill boxes linked to a web-based adherence platform, to create a differentiated care response to patient adherence and improve TB care among adult pulmonary DS-TB participants.

## Objectives

The primary objective of the trial is to evaluate whether implementation of daily adherence monitoring using either smart pill box or medication label, with differentiated response to patient adherence, decreases the proportion of adult pulmonary DS-TB participants with unfavourable outcomes (defined as poor end of treatment outcome or recurrence up to 6 months after the scheduled end of treatment) compared with the standard of care. The secondary objectives are to describe longitudinal DAT engagement and fidelity to adherence tools, project the epidemiological impact of scale-up of the interventions, estimate the cost-effectiveness and explore the feasibility and acceptability of implementing the interventions.

## Methods/Design

### Study design

This is a pragmatic three-arm cluster randomised trial (CRT) with the health facility as the unit of randomisation. A total of 78 health facilities from two regions are included in the trial. Health facilities were randomised (1:1:1), using stratification and restriction, to either the (i) smart pill box or (ii) medication label, both with daily monitoring and differentiated response to patient adherence, or (iii) standard of care.

### Study setting

Ethiopia is administratively divided into ten regional states and two chartered cities. The trial will be conducted in Addis Ababa city (the capital of Ethiopia) and Oromia region. These geographical settings were chosen in consultation with the National TB Programme (NTP) and the health facilities were selected in collaboration with NTP and the respective Regional Health Bureaus. The study health facilities include both large and small, urban, and rural facilities.

### Study population

All adult pulmonary DS-TB participants in the selected 78 health facilities who fulfil the following inclusion criteria will be invited to participate: aged 18 years or over, expecting to stay in the study area for at least 12 months, not requiring hospitalization, and providing consent. All who consented will be followed up for 6 months after treatment completion.

### Intervention

Participants in facilities randomised to either of the two intervention arms will be offered a smart pill box or medication label linked to the ASCENT web-based adherence platform for treatment adherence monitoring.

### Intervention arm 1: smart pill box

Participants in this arm will receive a smart pill box (evrimed 1000c), known also as Medication Event Reminder Monitor System (MERM). Dosing instruction and anti-TB drugs will be placed inside of the box. The box enables a configurable audio-visual reminder at a pre-defined time based on participants’ preference. When participants open the box, the embedded electronic device sends a signal in real-time to automatically log their daily dose to the ASCENT web-based application via a built-in mobile internet connection. If connectivity is temporarily unavailable, opening events are stored in the device memory and uploaded automatically when internet connectivity resumes. This allows the TB care provider to use the ASCENT adherence data platform to evaluate daily dosing and offer differentiated care (Table 1) as appropriate.

**Table 1:** Differentiated care response to patient adherence in intervention arms

Trigger	Intervention
Dose recorded (daily)	Medication label: message confirmation from patient of recorded dose Smart pill box: box opening (sound and/or light)
Patient missed daily dose (1 day)	Same day: patient receives a reminder SMS at 3 pm* Next day: HCW will receive a notification of patients with a missed dose on their Task List Follow-up actions: phone call to patient or treatment supporter, unless HCW is aware of a network issue**
Missed daily dose (2 consecutive days)	Same day: patient receives a reminder SMS at 3 pm* Next day: HCW will receive a notification of patients with a missed dose ion their Task List Follow-up actions: phone call to patient or treatment supporter**, unless HCW is aware of a network issue***
Patient persistently missed doses (≥ 5 consecutive doses or multiple times)***	Escalation to community health worker for home visit using a standardised set of messages and counselling
Patient persistently missed doses (> 14 consecutive doses or multiple times missing 7 doses)	Discontinuation from DAT intervention and switch to daily DOT under the HCWs observation for TB patients with persistent non-adherence will be considered

*SMS* short message service; *HCW* health care worker

*Among patients with a mobile phone; **If daily DOT resumes as the standard of care, phone calls will be replaced with escalation to community health worker for home visit; *** among patients with mobile phone access (either their own or treatment supporter)

### Intervention arm 2: medication labels

Participants in this arm will have a label, containing a code unique to a 1-week period, placed on their fixed-dose blister-packaged TB medication. When their daily dose is taken, participants will message this code using a toll-free text, which automatically logs their daily dose to the ASCENT web-based application. With the adherence data collected to the platform, the same procedure is used as for intervention arm 1 for offering differentiated care (Table 1), as appropriate. Participants who do not have access to a mobile phone will be given a smart pill box.

Originally the medication sleeve approach as used in the Uganda stepped-wedge trial [19] was planned, but due to frequent changes in the dimensions of the packaging used for TB medication which was also difficult to forecast, the label approach was adopted instead.

### Standard of care

Participants in the control arm, will utilize the standard of care for TB treatment support in the facilities randomized to this arm. During the intensive phase of treatment, this entails direct observation of medication intake by a health worker or TB treatment supporter, at a health facility (hospital, health center or health post), at patient’s workplace, residence institution or home. During the continuation phase, medication intake is under direct observation by a TB treatment supporter or through self-administration upon consultation with the TB provider. Missed clinic appointments should result in a phone call to the patient and missing two consecutive clinic appointments should prompt a home visit by a health care worker (HCW).

### Differentiated care delivery based on adherence to treatment

Participants in the intervention arms will have their daily adherence data from their DAT recorded on the ASCENT adherence platform.

TB care providers can remotely access and review their participants adherence data through the ASCENT adherence platform mobile Android app. Through this app, the TB care providers can see the day of treatment and medication taking history for patients on treatment for TB at their facility. It has built in data analytics to easily visualize and identify which participants have not taken their daily dose on a patient level dosing calendar. TB care providers offer interventions according to the number of doses a patient has missed. Table 1 summarizes the activities related to differentiated care response.

### Randomization

Stratified, restricted randomisation of health facilities was used to ensure reasonable baseline balance between the intervention arms and standard of care arm [33]. Stratification was on region and treatment outcomes in 2017–2019 (from the NTP TB registers), which divided the study facilities into four groups. The restrictions further considered data from all study facilities’ NTP TB registers and included percentage with poor treatment outcome; number of DS-TB registrations; HIV prevalence among DS-TB and urban/rural facility.

### Trial outcomes

The primary endpoint is a composite unfavourable outcome, similar to previous TB treatment trials [34–36] and the DAT intervention being evaluated in China [37], defined as either a (i) poor end of treatment outcome (lost to follow-up, death or treatment failure or (ii) treatment recurrence measured 6 months after the scheduled end of treatment). The secondary outcomes are:

Among adult pulmonary DS-TB participants in all arms:

(i) Poor end of treatment outcome (defined as above).
(ii) Lost to follow-up during treatment.

Among adult pulmonary DS-TB participants in intervention arms

(i) Longitudinal technology engagement with the DAT.
(ii) Fidelity to the DAT (includes the number of messages sent to participants and HCWs, number and length of phone calls to and from participants, number of health facility visits by participants and number of home visits to participants as well as describing device failures, inability to engage with the technology and cell phone access).

TB patients enrolled in the trial TB care providers and key stakeholders will also be interviewed to explore the institutional feasibility and acceptability of implementing the DAT.

### Impact modelling

The trial results will be used to inform a *Mycobacterium tuberculosis* transmission model for Ethiopia, which will be used to project the expected long-term epidemiological impact if the interventions were to be scaled up across the country. This model will include treatment pathways to calibrate DS-TB incidence and mortality in Ethiopia, including a detailed description of the treatment pathway and implementation of DAT. The model outcomes will include the impact of the interventions on TB deaths and cases averted, as well as TB patient costs averted through a potential reduction in clinic visits required.

### Economic evaluation

The effectiveness, utilisation, and cost data collected during the trial will be used to estimate the cost-effectiveness of the (i) smart pill box with differentiated care and (ii) medication label with differentiated care when compared against the standard of care for DS-TB patients in Ethiopia. A societal perspective will be taken in the analysis, estimating the cost to the provider of providing the service plus the costs incurred by patients in using the service. Provider costs will include the above service-level costs, specifically the costs of training and supporting the implementation of the intervention. Since TB disproportionately affects patients of lower socio-economic status, TB prevention through effective treatment adherence may change how TB is distributed across socioeconomic strata in a population. Using the results of the trial differentiated by socioeconomic status, we will quantify trade-offs related to health inequality in the decision to implement DATs by conducting a distributional cost-effectiveness analysis [38].

### Sample size calculation

Using facility-level TB treatment outcome data from 2018 in the ASCENT facilities, overall poor treatment outcome was observed to be 17% which was higher than the overall proportion reported in Ethiopia in 2017. Since our study will also capture recurrence 6 months after the end of treatment, we have assumed the percentage with unfavourable outcome varies between 17 and 20%.

Assuming 26 facilities per arm (78 in total), a harmonic mean of 50 TB patients across facilities, a type I error of 5%, percentage with unfavourable outcome in the standard of care of 17% and 20%, the trial will have 80 and 85% power to detect a one third reduction in the unfavourable outcome with a coefficient of variation (k) of 0.35 and k ≤ 0.3, respectively. We anticipate enrolling a total of around 4000 participants from all study facilities. All adult patients diagnosed with pulmonary DS-TB will be asked to participate in our study.

### Study procedures

All patients visiting the TB clinic of the health facilities will be screened for eligibility (criteria described in the study population) by the TB care provider, and if eligible, will be offered enrolment in the ASCENT study and followed by written informed consent (see Additional file 1 for informed consent form and patient information sheet). Among those who have consented, a brief socio-demographic case report form (CRF) will be filled out with the patient by the health care provider (as trained by the study team). In addition, contact details will also be collected to implement the intervention in the two intervention arms and to facilitate follow-up 6 months after the end of treatment in all three arms (see Additional file 2 for completed SPIRIT checklist).

All participants will be treated according to Ethiopian NTP guidelines, which is a fixed dose combination of isoniazid, rifampicin, ethambutol, and pyrazinamide for the 2-month intensive treatment phase, followed by isoniazid and rifampicin for the 4-month continuation phase [28]. At treatment initiation, all participants receive basic counselling on TB by the health facility staff according to routine practice, to improve their understanding of their illness, including the need for daily medication for the full 6-month duration of their treatment. Prior to patient enrolment, staff from both intervention facilities and standard of care facility will be invited to training that will standardize the information given to participants regarding TB disease and the importance of adherence. In addition, setting-appropriate information booklets will be provided to TB participants in intervention arms and posters for health facilities in all three arms.

Participants in intervention arm 1 (smart pill box) will be given a smart box, 14 days of medication, and a DAT booklet placed inside the smart box. At each opening, the box sends a signal to ASCENT platform and a daily digital log will be kept. Participants are requested to bring the box every 2 weeks during their 1st month of treatment and monthly thereafter for refill and return the box after treatment completion, i.e., 6 months after treatment initiation. Participants in intervention arm 2 (medication label) will be provided with 14 days of medication, a medication label with a number code, and a DAT booklet. The same refill schedule is used as for intervention arm 1 (smart pill box). Participants will be asked to send the number written on the medication label via short message service (SMS) to a standard number printed on the label every day after taking their daily dose. In the intervention arms, adherence data will be collected via the DAT on the ASCENT adherence platform, and TB care providers will use these data to offer patient-centred care. Participants in the intervention arms will also be offered educational and motivational messages through SMS. In addition, TB care providers will assist the participants to resolve any problems related to the DAT, as necessary. Participants in the intervention arm who decline to participate and participants in the standard of care arm will follow the standard care for TB treatment supervision as per the NTP (Table 2).

**Table 2:** Summary of activities in the intervention and standard of care arms

Activities	Intervention arms	Standard of Care arm
Counselling for TB adherence at treatment start	Patient education on adherence counselling will be provided as per standard of care	Patient education on adherence counselling will be provided as per standard of care
Registration and informed consent [research activity]	Adult patients satisfying the inclusion criteria and who consent to be enrolled into the study will provide written informed consent Patient will be registered on ASCENT adherence platform and upon registration receive confirmation verbally and/or by text message	Adult patients satisfying the inclusion criteria and who consent to be enrolled into the study will provide written informed consent
Data collection for the socio-demographic case report [research activity]	Tool delivered by the HCW	Tool delivered by the HCW
Explain DAT as a support tool for intervention	Delivered by HCW (standardised script) and pictorial booklet	Not applicable
TB treatment supervision	Self-administration of TB medication with support of DAT	As per standard of care (DOT at health facility or patient’ home or workplace, or self-administration)
TB medication provision	As per standard of care	As per standard of care
Daily dosing reminder	A reminder message to patient will be sent if a dose was not recorded on the platform* Depending on patient preference, the smart pill box can also remind participants using LED and/or sound	No reminder
Follow-up visits for treatment	Participants will be provided a return date to visit the health facility for refill	As per standard of care in the facility
Follow-up visit for treatment reminders	Depending on the DAT-, patient- and HCW preferences, participants can receive a reminder for follow-up visit via text message* or via smart pill box	No reminder
Adherence data	Information on adherence will be collected via the DAT and available via the ASCENT adherence platform to health HCW	As per standard of care (pill counts, patient treatment cards, etc.). Daily adherence data will only be available from those undergoing the facility-based DOT
Follow up visit during treatment	HCWs have access to the ASCENT adherence platform and will use the patient’ adherence calendar for counselling Routine sputum collection at 2, 5 and 6 months	As per standard of care in the facility Routine sputum collection at 2, 5 and 6 months
Education and motivational messages	Participants can receive periodic educational and motivational messages*	None
Telephone follow up 6 months after the end of treatment	Participants will be telephoned by research staff and a brief questionnaire conducted to ascertain if TB treatment has been restarted since the end of treatment* (among those eligible)	Participants will be telephoned by research staff and a brief questionnaire conducted to ascertain if TB treatment has been restarted since the end of treatment* (among those eligible)
Follow up visit 6 months after the end of treatment for sputum collection	Participants will be reminded of their appointment 2–3 days before the scheduled date (among those eligible). On attendance at the facility (same as the enrolment facility) sputum will be collected by the HCW for culture	Participants will be reminded of their appointment 2–3 days before the scheduled date (among those eligible). On attendance at the facility (same as the enrolment facility) sputum will be collected by the HCW for culture

*Among patients with a mobile phone

As per the Ethiopian NTP guidelines [28], sputum will be collected at the 2nd and 5th month of treatment to monitor treatment response, and at 6th month upon treatment completion to determine treatment outcome. Treatment outcome data will be abstracted from the Unit TB Register. Participants who had an end of treatment outcome of cured or completed treatment will be contacted by the study team via telephone around 6 months after treatment completion to check whether treatment for TB has been restarted. Participants who were bacteriologically confirmed pulmonary DS-TB at treatment initiation will also be invited to attend the facility to give a sputum for culture to determine their TB disease status at 6-months post treatment. Participants will be reimbursed as compensation for their time and travel expenses.

Adverse consequences of study participation include a potential risk of inadvertent disclosure of TB status by possession of DATs associated with TB treatment and receipt of SMSs relating to TB treatment. Adverse events (AEs) due to inadvertent disclosure of a participant’s TB status will be monitored across study arms. Data will be captured through both (i) participant reports to HCWs at the facility level, and (ii) researchers asking participants at the telephone interview 12 months from enrolment. AEs disclosed by participants to facility HCWs will be recorded on facility social harms register.

Participation in the study is voluntary and can be halted at any time. All participants will continue to receive routine care even if they withdraw from using the DAT or the study but will be asked if their adherence and treatment outcome data as well as their status 6 months after treatment completion can be collected.

### Data management

ASCENT Ethiopia data will originate from three different sources: the ASCENT adherence platform, ASCENT research database and ASCENT participant identifiers database. For the ASCENT adherence platform, all patient identifiable data will be stored according to Ethiopian regulations related to storage of identifiable data. The ASCENT adherence platform will be hosted on the Everwell Hub, a hosted web patient management application. The platform is used by TB care providers to review patient medication adherence, track SMS communication with patients and, document adherence support actions. As part of privacy protection, user authentication and hierarchical user authorization are applied for accessing the ASCENT platform in addition to obtaining initial agreement from the patients. The Everwell Hub resides primarily on a Microsoft SQL database server and web servers located securely in the cloud.

The ASCENT research database and participant identifiers database will be hosted in-country at KNCV Tuberculosis Foundation Ethiopia office, using REDCap [39], a secure web application for building and managing online surveys and databases.

### Trial governance

A Technical Advisory Group (TAG) has been set up to provide oversight, monitor and oversee progress for the Ethiopia cluster randomized trial and companion studies in the Philippines, South Africa, Tanzania, and Ukraine. The TAG meets every 6 months and is composed of representatives from the five countries and chaired by a senior researcher in Uganda.

A Community Advisory Board (CAB) has been established in Ethiopia for the ASCENT project to oversee the research as well as all ASCENT activities in Ethiopia. The CAB’s main role will be to advise on study messaging (support texts and reminders), the differentiated care plan and enhance communication between researchers and study community members.

A Trial Management Group meets regularly to oversee the day-to-day activities of the research. The group includes the Trial Manager, representatives from the NTP in Ethiopia, LSHTM, KNCV Ethiopia, and KNCV the Netherlands. A representative from the Community Advisory Board will also attend the meeting once every 2 months. The Trial Management Group is responsible for the trial design, leading the implementation, data analysis, publication, and determining authorship eligibility, site training, study monitoring, and quarterly site visits.

### Statistical analysis plan

Analyses of primary and secondary outcomes will follow intention-to-treat and per-protocol approach with methods appropriate for the clustered randomised trial design and will be based on two pair-wise comparisons of each intervention arm with the standard of care. The primary analysis will employ an individual-level logistic regression model with random effects (to account for within-cluster variation) to estimate the intervention effect (odds ratio) and associated 95% confidence interval, adjusting for randomisation strata. Further adjustment for patient-level covariates showing imbalance by study arm will be considered. Subgroup analyses will be conducted for the primary outcome and include urban/rural, gender, literacy levels, socio-economic status, and type of health care provider. A full statistical analysis plan will be developed prior to the end of data collection.

### Ethical consideration and dissemination

The trial has approval from London School of Hygiene & Tropical Medicine Ethics Committee (19120 ‑ 1), United Kingdom; WHO Ethical Review Committee (0003297), Switzerland; Addis Ababa City Administration Health Bureau Public Emergency and Health Research Directorate Institutional Review Board (A/A/H/B/1/978/227), and Oromia Regional Health Bureau Public Emergency and Health Research Directorate Institutional Review Board (BEFO/HBTFH/1-16/10322), Ethiopia. Written informed consent from individuals to participate in the main effectiveness study will be collected by a TB care provider at the health facility. For the sub-studies, informed consent will be sought by research staff prior to interview. A unique study identifier will be assigned to each individual who has consented to the study. Anonymized individual-level data with only the unique study identifier will be visible to enable project/research staff to monitor study activities and evaluate fidelity to the intervention. All databases will be secured with password-protected access systems. All paper records will be stored at the participating clinics in locked filing cabinets and access to the records will be restricted to specified study team members. Any study related documents will be identified using the participant’s study number only, with locator information stored separately and available only to facility staff and research staff contacting participants at 12 months. All identifiers are stored in a database with password-protected limited access.

The primary results from the main trial will be presented to national stakeholders, and disseminated to the Community Advisory Board, stakeholders, and participants through meetings and policy brief. Findings from the trial will also be presented at conferences and published as articles for suitable scientific journals. All de-identified research datasets will be shared for the sake of transparency and reproducibility. Data sharing agreements will be developed taking into account Ethiopian-specific data sharing regulations and to prioritize work led by researchers in Ethiopia.

Major changes to the study will be updated in the protocol and trial registration, reported to the ethics committees for approval, and communicated to the CAB and TAG without delay.

### Sub-studies

We will conduct a total of four sub-studies, of which three will be conducted in selected facilities participating in the trial, among a subgroup of pulmonary DS-TB participants, HCWs and key stakeholders. In sub-study 1, patient and facility-level acceptability and cost survey data will be collected. Sub-study 2, using qualitative methods, will focus on exploring patient experiences of taking TB treatment using a DAT. In sub-study 3, a qualitative study of the acceptability and feasibility of DAT will be conducted through interviews with health care workers and key stakeholders. These sub-studies will contribute to the process evaluation of the interventions that will inform program implementation. The fourth sub-study will focus on enrolling DR-TB participants from DR-TB treatment facilities to measure acceptability of the DAT systems (smart pill box and video supported treatment).

## Baseline assessment of the clusters in the trial

In collaboration with the NTP, a total of 78 health facilities from the two regions in Ethiopia have been identified to participate in the ASCENT project. All the 36 facilities from Addis Ababa were urban health centres. The health facilities in Oromia included five urban primary hospitals, five rural health centres and 32 urban health centres. An average of 63 (range 30–142 in Addis Ababa; 34–132 in Oromia) pulmonary TB patients in these facilities were started on treatment in 2018. Nearly 60% (Addis Ababa) and 52% (Oromia) of these patients had bacteriologically confirmed PTB.

## Preliminary findings from run-in phase

Due to the large number of clusters, a staggered approach over a 5-month period was employed to include facilities in the run-in phase. The study tools and activities to be employed during the research period were implemented and piloted to ensure full operationality of the intervention at the beginning of the research period. Participants enrolled during this phase will be followed up to the end of treatment. Over 1250 TB patients were screened for eligibility across all study arms and 586 enrolled, of which 40% are females, median age 30 years (interquartile range 23–40 years) and 67% had smear confirmed TB.

## Discussion

This trial is a pragmatic cluster-randomised trial and the first of its kind to evaluate effectiveness of smart-pill box and medication labels linked to a web-based adherence platform with differentiated response to patient adherence in Ethiopia. The aim of this trial extends beyond reducing the proportion of poor end of TB treatment outcomes to include evaluating the 6-month disease free survival, process evaluation, and the wider epidemiological impact and cost-effectiveness of DAT systems for DS-TB patients.

Previous trials evaluating the effectiveness of DAT on treatment outcomes have shown mixed results [18, 20–22]. Findings from trials in Pakistan, China and Cameroon suggested that text messaging reminders have neither improved medication adherence [21, 22] nor did they increase treatment success and cure proportions [18]. A trial in Ethiopia found mobile phone-based weekly refilling with daily medication reminder system improved medication adherence but with no significant effect on treatment success [23]. However, another trial from China indicated that text messaging positively impacted treatment completion rate and negatively influenced missed dose rate and interrupted treatment rate [20] and a trial conducted in Kenya assessing a text messaging intervention demonstrated a reduction in unsuccessful treatment outcome, mainly though reducing loss to follow-up [24]. Similarly, a cluster-randomised trial from China revealed reminders from medication monitors improved medication adherence in TB patients [21]. Finally, a stepped-wedge CRT trial in Uganda showed no impact of the medication sleeves DAT intervention on successful treatment outcomes in an intent to treat analysis (all patients initiated on TB treatment) though did demonstrate a three-fold increase in odds of successful outcomes in an analysis restricted to patients who did enrol onto the DAT in the intervention phase [19].

While RCTs are regarded as the “gold standard” in evaluating the specific interventions [33], the optimised study conditions make inference regarding their real-world effectiveness problematic [40]. Using the Pragmatic Explanatory Continuum Indicator Summary (PRECIS-2) tool [41] that measures trial design features, we argue that key aspects of our trial the manner of enrolment, eligibility criteria, delivery of the intervention (primarily implemented by health facility staff), measurement of some outcomes, and process evaluation are pragmatic, and our findings have more relevance to directly inform DAT scale-up in countries.

In countries around the world, patients and their providers face several challenges with facility-based DOTs [4–7]. The need for patients to visit a health facility on either a daily or a weekly basis places a significant burden on both the patient and the health system. Given the variety of patient needs to complete treatment, the traditional DOTs approach may not be sufficient to provide an adequate patient-centred model to support treatment. Thus, alternative approaches such as DATs to enhance current practices are needed.

DATs paired with an overarching adherence platform may help to overcome health care disparities by allowing patients greater freedom to self-manage and participate in their own care, reduce financial burdens, and restore autonomy and dignity, while simultaneously empowering providers with more reliable and timelier patient data to inform care decisions for those patients with the greatest need. In addition, such platforms allow TB programs to integrate and aggregate data generated by different types of DATs. Additional benefits to the health system could also be gained upon successful implementation of DATs at times of unprecedented global health threats that challenge the routine health care services. For instance, in the era of COVID-19 pandemic, the standard of care has changed, in particular DOT, and DATs have been widely recommended to be ensure adequate treatment and adherence support [42, 43]. However, DATs may be least accessed by the priority populations for whom they are intended, due to limited access to mobile phones [44], low literacy and social support [27], resulting in differential uptake to interventions based on the form of technology.

The ASCENT project has set primary and secondary outcomes, which are well characterized and relevant for TB management, and that allow for rigorous evaluation of the effectiveness and feasibility of DAT systems to improve disease-free survival among adult pulmonary TB participants in Ethiopia. Furthermore, the costing component of the ASCENT project enables us to analyse the cost-effectiveness of the intervention within the trial population as well as the patient costs averted. By implementing this project to improve adherence, we hope to generate evidence to improve treatment completion and reduce rates of loss to follow-up, while reducing TB patient costs. If successful, this evaluation will generate further evidence for optimal use and scale-up of DATs in Ethiopia and elsewhere.

### Trial status

The protocol is version 2.1.1, dated 31 March 2021. The trial started recruitment to the run-in phase on December 23, 2020. Recruitment to the main trial started in June 2021 and will continue for 12 months.

## Supplementary Information

**Additional file 1. **Informed consent form and patient information sheet.

**Additional file 2. **Completed Spirit checklist.

## Data Availability

After publication of the main trial results, there will be a period of exclusive access to the data for researchers from the ASCENT consortium and local research community in each participating country. After the period of exclusive use, de-identified data will be made available to users outside of the ASCENT team and the local research community via a publicly available data repository.

## Abbreviations

CRT: Cluster randomised trial
DAT: Digital adherence technology
DOTS: Directly observed treatment short-course
DOT: Directly observed treatment
DS: Drug-sensitive
NTP: National TB Programme
SDG: Sustainable Development Goals
SMS: Short message service
TB: Tuberculosis
WHO: World Health Organisation
