# Supplementary material for: Evaluation of implementation and effectiveness of digital adherence technology with differentiated care to support tuberculosis treatment adherence and improve treatment outcomes in Ethiopia: a study protocol for a cluster randomised trial
Source: BMC Infect Dis. 2021 Nov 10;21:1149. doi: 10.1186/s12879-021-06833-x (PMC8579414; doi:10.1186/s12879-021-06833-x)
Supplement: Supplementary file 1 — Additional file 1. Informed consent form and patient information sheet. [file 12879_2021_6833_MOESM1_ESM.docx]

**PARTICIPANT Information Sheet and CONSENT Form for ETHIOPIA
Effectiveness evalUation – INTERVENTION (SMART pill box)**


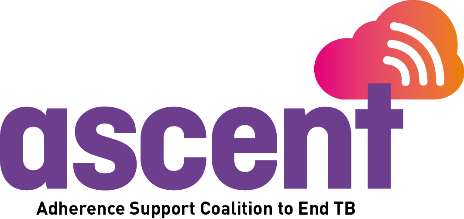


**STUDY TITLE: Adherence Support Coalition to End TB: evaluation of digital adherence technologies for TB treatment in Ethiopia**

**SHORT TITLE: ASCENT-effectiveness evaluation**

**SPONSOR: KNCV Tuberculosis Foundation**

**PRINCIPAL INVESTIGATOR: Katherine Fielding**

**INSTITUTION: London School of Hygiene & Tropical Medicine**

**COUNTRY PRINCIPAL Taye Letta**

**INVESTIGATOR:**

**INSTITUTION: National TB Programme in Ethiopia**

**DAYTIME HOURS TELEPHONE NUMBER(S): +251-911-367284**

**To the potential participant:** This consent form may contain words that you do not understand. Please ask the study staff to explain any words or information that you do not clearly understand. You may take home an unsigned copy of this consent form to think about or discuss with family or friends before making your decision.


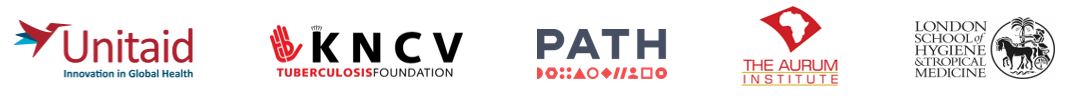


Hi, my name is ……………………………….. (Name of staff taking consent) and work as a …………………….. (Job title) at ……………………………….. (Health facility).

We would like to invite you to take part in a research study entitled “*ASCENT effectiveness evaluatio*n”.

1. Before agreeing to take part in this study, it is important that you read and understand (or have read to you) what is expected of you if you take part.
2. If you have any questions do not hesitate to ask me.
3. You should not agree to take part unless you are happy with all the things that may be asked of you.
4. If you agree to take part in this study, you will be asked to sign/thumbprint this document to confirm that you understand the study. You will be given a copy to keep.

**Why are we doing the study?**

- Research is a way to learn the answer to a question and use the answers to improve health care services.
- “TB” is a shorter way of saying tuberculosis, which is spread through coughing/ sneezing the TB germs.
- People with TB have to take treatment every day for 6 months and this can be very hard to do.
- Some people with TB have difficulties remembering to take their TB treatment everyday on time.
- This research study is part of a larger project called ASCENT and will be looking at ways digital adherence technologies may help people take their TB treatment.
- Some people with TB in this study will be given “digital adherence technologies” which are designed to help remind people to take their TB treatment daily and on time.
- You will be given one of these technologies to support you in your TB treatment. Every time you use the digital adherence technology to take your treatment, your doctor or health care provider will receive an automatic message that you took your daily TB pills.
- Sending this information about your TB pill-taking to your health care worker can help them to determine the best treatment approach for you and provide you with support if you need it. Even if you are using the digital adherence technology, you will still receive care from your health care worker.
- This study will also look at patients being cared for in the usual way by clinic health care workers.

There are three different parts (groups) to this study: smart pill box INTERVENTION, medication label INTERVENTION and STANDARD OF CARE.

The STANDARD OF CARE group: this means the usual standard of care is used to help patients like yourself to take TB treatment.

Smart pill box INTERVENTION group: people are given a smart pill box (called evriMED) when they start TB treatment.

Medication label INTERVENTION group: people are given their treatment in customized packaging such as special envelopes or labels with a code to register your dose.

Your clinic is in the **smart pill box INTERVENTION group**

In this INTERVENTION group you will be given a smart pill box (sometimes called evriMED) and when you receive your medication at the health facility it will be put inside the pill box. The smart pill box is provided to you for free, but you will need to return it at the end of your treatment for TB. Every time you open the smart pill box, the electronic device inside the box sends a small signal (similar to an SMS or text) to your health care worker’s computer to record that you have opened the box to take your pills. Sending information about your pill-taking to your health care provider will help them to determine the best treatment approach for you.

The smart pill box will remind you (with a flashing green light or beeping sound) to take your medication daily and on time and not to miss any doses. There is also information on the box to help remind you of how to take your treatment. If you forget to take a dose your health care worker may either call, send you a message or visit you and offer help if you are having problems taking treatment.

The smart pill box only sends information about when the box is opened, how many times it is opened, and what the battery level of the device is. It is not able to not record or send any information about your family or your home. The smart pill box has a battery that lasts for several months and can be charged using the charging cable or at the clinic if needed.

**Why are we asking you to help?**

We are asking you to help in this research since you are to be treated for TB at this facility that is using digital adherence technologies to assist patients like yourself to take their TB treatment.

**How long do you have to be part of the study?**

- The study will take place in 78 clinics in 2 districts in Ethiopia
- The participants will include approximately 4000 adults.
- The total amount of time required for your participation in this study will be a maximum of 12 months.

**What will happen if you take part in this study?**

Please remember that your participation is voluntary and you:

1. May refuse to participate in the study
2. Have the right to stop taking part in the study at any time.

If you agree to take part in this study, this is what will happen:

- We will ask you some questions about yourself (for example, age, address, your education). This talk may last about 5 minutes. You do not have to answer questions if you do not want to.
- Your health care worker will demonstrate how the smart pill box works
- You will be provided with the box and a booklet that explains the use of the smart pill box
- During treatment registration some information that is written on your treatment card by the health worker, such as your phone number and TB registration number, will also be stored in a computer or tablet where only others who help in your treatment at the facility or who work on this study can see it.
- During your treatment, on a daily basis, the technology will send a signal to your health care worker if you took your medication.
- You and your health care worker may look at your data to support you taking TB medicine. As part of the study, you may receive text messages to the phone number you register, such as reminders or motivational messages. Therefore, if you do not have your own phone, you are encouraged to use a phone you share with people you can confide in and who can support you.
- The smart pill box is provided to you for free for your use during treatment. You will not be asked to pay for anything. You will return the box to the health facility once you have completed your treatment.
- In around 12 months time a research assistant will contact you directly, using the phone number you give us, taking care to be sure we are talking to you before we ask questions about your health. They will ask you whether you have had to restart TB treatment and whether anyone has found out, by accident, you are on TB treatment. This talk may last about 5 minutes.
- Depending on the type of TB you have, you may also be asked to attend this clinic to give a spit (sputum) sample to check that the TB has not come back. We will reimburse you 85ETB for travel to the clinic.
- We request your permission to access your medical records and laboratory results from this clinic or any other clinic where you sought care.
- There is no cost to you for taking part in the study.
- You will **NOT** be paid to take part in this study

**Use of your information**

- To be able to understand how these digital technologies may help people like yourself take and complete their TB treatment, researchers will be able to see your pill-taking information - when you open the box and what sorts of help such as SMS, phone call and home visit you have received.
- This information will be used to write report or article about how these digital technologies can be used to improve the care given to TB patients like you around the world.
- This information will be anonymized, meaning that the researchers will never see your name, your address, or any other personal information that identifies you or your family with your pill-taking information.
- At the end of the project, study data will be made available to other researchers worldwide for non-commercial use to improve medical knowledge and patient care. Your study data will only refer to a study number and will not include your name, phone, address or any other personal identifier. While we believe the risk of someone identifying you is very small, we will take every precaution to ensure that other data elements such as the name or location of the health facility that could be used to identify you, will be modified or removed from the study data.

**What are the risks of participating in the study?**

- Some people may feel anxious or uncomfortable using these new technologies. These digital adherence technologies may be more “visible” to others in your home or workplace and may result in someone finding out that you are on TB treatment, by accident.
- You may reduce the chance of others guessing that you may have TB by keeping the smart pill box in a bag as you travel. Some people may feel anxious or uncomfortable answering some questions, but you may skip any question that you are not comfortable with and you may stop the interview at any time.
- Friends and family members may react by becoming worried that you joined the study without consulting them.

**What are the benefits of participating in the study?**

- These digital adherence technologies may help you as patient to take your daily medication at a time and place that suits you best.
- A possible benefit from this new approach could mean that the frequency of your visits to the health facility will be lower than that for other TB patients for whom this is the only option.
- The technologies provide recent information on your pill-taking to the TB doctor or nurse to help them determine the most appropriate treatment approach for you. This way they can use this information to work with you to help make sure that you complete your treatment as intended to cure you of TB.
- Lastly, the information you provide and experience you have from using these technologies will be used to help improve the success of treatment for TB in patients like yourself.

**What happens if you do not agree to take part in this study?**

- If you do not want to be part of this study, simply say so.
- If at any time after signing up to take part in the study, you decide that you want to stop using the smart pill box, you may do so. We would like to still access your medication records and speak to you 12 months after starting TB treatment – we will ask if this is ok with you. You will continue to receive TB treatment by the health care workers at this health facility.
- If at any time, you also decide to stop sharing your information, you may do so. We will delete your data.
- Stopping the study will not affect the kind of treatment you receive. You will continue to receive TB treatment by the health care workers at this health facility.

**How is the information collected during this study going to be kept confidential?**

- All information about you and your treatment collected during this study will be kept secure and confidential (kept secret). The data on the computer is kept on a secure server, encrypted and password protected. For research staff, your personal information (such as your name) is kept separate from other information you provide, in a password protected database. Actual responses to questions will only be identified using a study number that only the study staff can trace back to you.
- Reports about the study may be made to the government or the funder, but you will not be personally identified in any report about this study.
- In addition, we may be asked to provide data to the funder but your personal identifying information will not be included in that data.
- Any data reported in scientific journals will not include any information that identifies you as a participant in this study.
- The information might also be inspected by the Public Emergency and Health Research Directorate Institutional Review Boards of the Addis Ababa City Administration Health Bureau and Oromia Regional Health Bureau Public Emergency and Health Research Directorate Institutional Review Board to insure the data are safe and your privacy is being respected.

If you are willing to participate to use the digital adherence technology to support your TB treatment, and sharing the information collected using it, you will have to sign the consent form below. You can ask us questions about anything before deciding to participate or not.

**Study discontinuation**

If at any time the study is discontinued by the funder or any of the relevant governing bodies, you will continue to receive treatment at this health facility according to the standard of care.

**Ethical approval**

This study protocol has been submitted to the Public Emergency and Health Research Directorate Institutional Review Boards of the Addis Ababa City Administration Health Bureau and Oromia Regional Health Bureau Public Emergency and Health Research Directorate Institutional Review Board and written approval has been granted by that committee. The study has been structured in accordance with the **Declaration of Helsinki** (last updated: October 2013), which deals with the recommendations guiding doctors in biomedical research involving human participants. A copy may be obtained from me should you wish to review it. The study is paid for by Unitaid.

If you have any further questions or queries about this project, please do not hesitate to contact Dr Gedion Tefera (ASCENT Country Project manager) on +251-0116630146 or Katherine Fielding, London School of Hygiene & Tropical Medicine, UK. (e-mail: [Katherine.fielding@lshtm.ac.uk](mailto:Katherine.fielding@lshtm.ac.uk)) OR you may also telephone Dr Amare W. Tadesse (ASCENT country Trial Manager) on +251-0116630164 during office hours.

**PARTICIPANT QUESTIONS?:**

Did the participant raise any questions?

YES/NO

If YES- What where they:

**(This information sheet will be available in local languages - Amharic and Afaan Oromootiin)**

- I, hereby confirm that I have been informed by ………………..………..about the nature, conduct, benefits and risks of the study (*ASCENT-effectiveness evaluation*).
- I have received, read and understood the above written information (participant Information leaflet and Informed consent) regarding the study.
- I am aware that the results of the study, including personal details regarding my sex, age, date of birth, initials and diagnosis will be anonymously processed into a study report.
- I may, at any stage, without prejudice, withdraw my consent and participation in the study.
- I have had sufficient opportunity to ask questions and (of my own free will) declare myself prepared to participate in the study.

**PARTICIPANT**

________________________________ ___________________________________

Participant’s Name (print) Participant’s Signature

Date: __________________________

I,………………………………(insert name of research staff), herewith confirm that the above participant has been fully informed about the nature, conduct and risks of the above study.

**HEALTH CARE WORKER**

____________________________ ___________________________

Study Staff Member (print) Study Staff Member’s Signature

Date: _____________________________

**WITNESS* (If applicable):**

______________________ ___________________________

Print Name Signature:

Date: _____________________________

Contact details:

Trial Manager: Dr Amare W Tadesse +251-0116630164

Country Investigator: Taye Letta +251-911367284.

**PARTICIPANT Information Sheet and CONSENT Form for ETHIOPIA
Effectiveness evalUation – INTERVENTION (MEDICATION LABEL)**


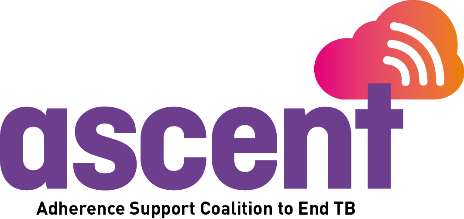


**STUDY TITLE: Adherence Support Coalition to End TB: evaluation of digital adherence technologies for TB treatment in Ethiopia**

**SHORT TITLE: ASCENT-effectiveness evaluation**

**SPONSOR: KNCV Tuberculosis Foundation**

**PRINCIPAL INVESTIGATOR: Katherine Fielding**

**INSTITUTION: London School of Hygiene & Tropical Medicine**

**COUNTRY PRINCIPAL Taye Letta**

**INVESTIGATOR:**

**INSTITUTION: National TB Programme in Ethiopia**

**DAYTIME HOURS TELEPHONE NUMBER(S): +251-911-367284**

**To the potential participant:** This consent form may contain words that you do not understand. Please ask the study staff to explain any words or information that you do not clearly understand. You may take home an unsigned copy of this consent form to think about or discuss with family or friends before making your decision.


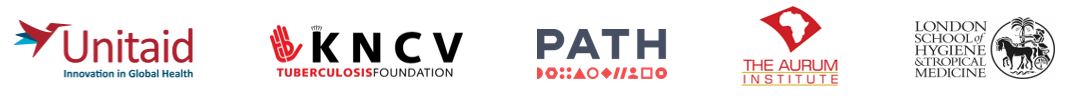


Hi, my name is ……………………………….. (Name of staff taking consent) and work as a …………………….. (Job title) at ……………………………….. (Health facility).

We would like to invite you to take part in a research study entitled “*ASCENT effectiveness evaluatio*n”.

1. Before agreeing to take part in this study, it is important that you read and understand (or have read to you) what is expected of you if you take part.
2. If you have any questions do not hesitate to ask me.
3. You should not agree to take part unless you are happy with all the things that may be asked of you.
4. If you agree to take part in this study, you will be asked to sign or thumbprint this document to confirm that you understand the study. You will be given a copy to keep.

**Why are we doing the study?**

- Research is a way to learn the answer to a question and use the answers to improve health care services.
- “TB” is a shorter way of saying tuberculosis, which is spread through coughing/ sneezing the TB germs.
- People with TB have to take treatment every day for 6 months and this can be very hard to do.
- Some people with TB have difficulties remembering to take their TB treatment every day on time.
- This research study is part of a larger project called ASCENT and will be is looking at ways digital adherence technologies may help people take their TB treatment.
- Some people with TB in this study will be given “digital adherence technologies” which are designed to help remind people with TB to take their TB treatment daily and on time.
- You will be given one of these technologies to support you in your TB treatment. Every time you use the digital adherence technology to take your treatment, your doctor or health care provider will receive an automatic message that you took your daily TB pills.
- Sending this information about your TB pill-taking to your health care worker can help them to determine the best treatment approach for you and provide you with support if you need it.
- Even if you are using the digital adherence technology, you will still receive care from your health care worker.
- This study will also look at patients being cared for in the usual way by clinic health care workers.

There are three different parts (groups) to this study: smart pill box INTERVENTION, medication label INTERVENTION and STANDARD OF CARE.

The STANDARD OF CARE group: this means the usual standard of care is used to help patients like yourself to take TB treatment.

Smart pill box INTERVENTION group: people are given a smart pill box (called evriMED) when they start TB treatment.

Medication label INTERVENTION group: people are given their treatment in customized packaging such as special s or labels with a code to register your dose.

Your clinic is in the **medication label INTERVENTION group.**

In this INTERVENTION group people are given their treatment in customized packaging such as special s or labels (called medication label). There are printed instructions and pictures to help you remember how to take your medication pills. Every day you should send a free SMS/text message with this number code to the phone number that is printed on customized packaging. There is no charge for this text.

When you send the SMS/text, your health care worker will be automatically informed that you took your medication for the day. This helps them make sure you are staying on treatment so that you can become healthy again. Sending this information about your pill-taking to your health care provider will help them to determine the best treatment approach for you and will help them provide you with support when you need it. If you forget to take a dose your health care worker may either call, send you a message or visit you and offer help if you are having problems taking treatment.

**Why are we asking you to help?**

We are asking you to help in this research since you are to be treated for TB at this facility that is using medication label to assist patients like yourself to take TB treatment.

**How long do you have to be part of the study?**

- The study will take place in 78 clinics in 2 districts in Ethiopia
- The participants will include approximately 4000 adults.
- The total amount of time required for your participation in this study will be maximum of 12 months.

**What will happen if you take part in this study?**

Please remember that your participation is voluntary and you:

1. May refuse to participate in the study
2. Have the right to stop taking part in the study at any time.

- If you agree to take part in this study, this is what will happen. We will ask you some questions about yourself (for example, age, address, your education). This talk may last about 5 minutes. You do not have to answer questions if you do not want to.
- Your health care worker will show you how to use the medication label.
- You will receive your treatment in medication label and a booklet that explains how to use the medication label.
- There will be no additional costs for you to use the digital adherence technology during treatment. All SMS/text messages you send to the special number are free of costs and will not use any credit from your account.
- During treatment registration some information that is written on your treatment card by the health worker, such as your phone number and TB registration number, will also be stored in a computer or tablet where only others who help in your treatment at the facility or who work on this study can see it.
- As part of the study, you may receive messages to the phone number you register such as reminders or motivational messages. Therefore, if you do not have your own phone, you are encouraged to use a phone you share with people you can confide in and who can support you.
- You and your health care worker may look at your data to support you taking TB medicine.
- In around 12 months time a research assistant will contact you directly, using the phone number you give us, taking care to be sure we are talking to you before we ask questions about your health. They will ask you whether you have had to restart TB treatment and whether anyone has found out, by accident, you are on TB treatment. This talk may last about 5 minutes.
- Depending on the type of TB you have, you may also be asked to attend this clinic to give a spit (sputum) sample to check that the TB has not come back. We will reimburse you 85ETB for travel to the clinic.
- We request your permission to access your medical records and laboratory results from this clinic or any other clinic where you sought care.
- There is no cost to you for taking part in the study.
- You will **NOT** be paid to take part in this study

**Use of your information**

- To be able to understand how these digital technologies may help people like yourself take and complete their TB treatment, researchers will be able to see your pill-taking information - when you send a text message/SMS and what sorts of help such as SMS, phone call and home visit you have received.
- This information will be used to write report or article about how these digital technologies can be used to improve the care given to TB patients like you around the world.
- This information will be anonymized, meaning that the researchers will never see your name, your address, or any other personal information that identifies you or your family with your pill-taking information.
- At the end of the project, study data will be made available to other researchers worldwide for non-commercial use to improve medical knowledge and patient care. Your study data will only refer to a study number and will not include your name, phone, address or any other personal identifier. While we believe the risk of someone identifying you is very small, we will take every precaution to ensure that other data elements such as the name or location of the health facility that could be used to identify you, will be modified or removed from the study data.

**What are the risks of participating in the study?**

- Some people may feel anxious or uncomfortable using these new technologies. These digital adherence technologies may be more “visible” to others in your home or workplace and may result in someone finding out that you are on TB treatment, by accident.
- You may reduce the chance of others guessing that you may have TB by keeping the **medication label** in a bag as you travel.
- If other people have access to the phone that you register, they might see messages for you about your treatment. If you have a shared phone, please think carefully about whether you are comfortable with others possibly seeing these messages. If you do not feel comfortable using a shared phone, then you will be offered the smart pill box and review with your health care worker an information sheet and informed consent for smart pill boxes. Some people may feel anxious or uncomfortable answering some questions, but you may skip any question that you are not comfortable with and you may stop the interview at any time.
- Friends and family members may react by becoming worried that you joined the study without consulting them.

**What are the benefits of participating in the study?**

- These digital adherence technologies may help you as patient to take your daily medication at a time and place that suits you best.
- A possible benefit from this new approach could mean that the frequency of your visits to the health facility will be lower than that for other TB patients for whom this is the only option.
- The technologies provide recent information on your pill-taking to the TB doctor or nurse to help them determine the most appropriate treatment approach for you. This way they can use this information to work with you to help make sure that you complete your treatment as intended to cure you of TB.
- Lastly, the information you provide and experience you have from using these digital technologies will be used to help improve the success of treatment for TB in patients like yourself.

**What happens if you do not agree to take part in this study?**

- If you do not want to be part of this study, simply say so.
- If at any time after signing up to take part in the study, you decide that you want to stop using the medication label, you may do so. We would like to still access your medication records and speak to you 12 months after starting TB treatment – we will ask if this is ok with you. You will continue to receive TB treatment by the health care workers at this health facility.
- If at any time, you also decide to stop sharing your information, you may do so. We will delete your data.
- Stopping the study will not affect the kind of treatment you receive. You will continue to receive TB treatment by the health care workers at this health facility.

**How is the information collected during this study going to be kept confidential?**

- All information about you and your treatment collected during this study will be kept secure and confidential (kept secret). The data on the computer is kept on a secure server, encrypted and password protected. For research staff, your personal information (such as your name) is kept separate from other information you provide, in a password protected database. Actual responses to questions will only be identified using a study number that only the study staff can trace back to you.
- Reports about the study may be made to the government or the funder, but you will not be personally identified in any report about this study.
- In addition, we may be asked to provide data to the funder but your personal identifying information will not be included in that data.
- Any data reported in scientific journals will not include any information that identifies you as a participant in this study.
- The information might also be inspected by the the Public Emergency and Health Research Directorate Institutional Review Boards of the Addis Ababa City Administration Health Bureau and Oromia Regional Health Bureau Public Emergency and Health Research Directorate Institutional Review Board who have your safety and privacy in mind.

If you are willing to participate to use the digital adherence technology to support your TB treatment, and sharing the information collected using it, you will have to sign the consent form below. You can ask us questions about anything before deciding to participate or not.

**Study discontinuation**

If at any time the study is stopped by the funder, the Trial Advisory Group, or any of the relevant governing bodies, you will continue to receive treatment at this health facility according to the standard of care.

**Ethical approval**

This study protocol has been submitted to the Public Emergency and Health Research Directorate Institutional Review Boards of the Addis Ababa City Administration Health Bureau and Oromia Regional Health Bureau Public Emergency and Health Research Directorate Institutional Review Board and written approval has been granted by that committee. The study has been structured in accordance with the **Declaration of Helsinki** (last updated: October 2013), which deals with the recommendations guiding doctors in biomedical research involving human participants. A copy may be obtained from me should you wish to review it. The study is paid for by Unitaid.

If you have any further questions or queries about this project, please do not hesitate to contact Dr Gedion Tefera (ASCENT Country Project manager) on +251-0116630146 or Katherine Fielding, London School of Hygiene & Tropical Medicine, UK. (e-mail: [Katherine.fielding@lshtm.ac.uk](mailto:Katherine.fielding@lshtm.ac.uk)) OR you may also telephone Dr Amare W. Tadesse (ASCENT country Trial Manager) on +251-0116630164 during office hours.

**PARTICIPANT QUESTIONS?:**

Did the participant raise any questions?

YES/NO

If YES- What where they:

**(This information sheet will be available in local languages - Amharic and Afaan Oromootiin)**

- I, hereby confirm that I have been informed by ………………..………..about the nature, conduct, benefits and risks of the study (*ASCENT-effectiveness evaluation*).
- I have received, read and understood the above written information (participant information sheet and informed consent) regarding the study.
- I am aware that the results of the study, including personal details regarding my sex, age, date of birth, initials and diagnosis will be anonymously processed into a study report.
- I may, at any stage, without prejudice, withdraw my consent and participation in the study.
- I have had sufficient opportunity to ask questions and (of my own free will) declare myself prepared to participate in the study.

**PARTICIPANT**

________________________________ ___________________________________

Participant’s Name (print) Participant’s Signature

Date: __________________________

I,………………………………(insert name of research staff), herewith confirm that the above participant has been fully informed about the nature, conduct and risks of the above study.

**HEALTH CARE WORKER**

____________________________ ___________________________

Study Staff Member (print) Study Staff Member’s Signature

Date: _____________________________

**WITNESS* (If applicable):**

______________________ ___________________________

Print Name Signature:

Date: _____________________________

Contact details:

Trial Manager: Dr Amare W Tadesse +251-0116630164

Country Investigator: Taye Letta +251-911367284.

**PARTICIPANT Information Sheet and CONSENT Form for ETHIOPIA
Effectiveness evalUation – INTERVENTION (MEDICATION SLEEVE/label – switch to SMART PILL BOX)**


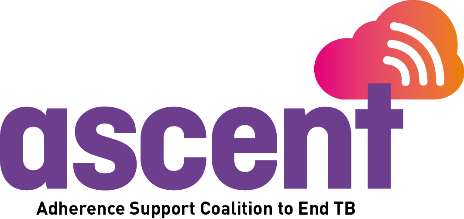


**STUDY TITLE: Adherence Support Coalition to End TB: evaluation of digital adherence technologies for TB treatment in Ethiopia**

**SHORT TITLE: ASCENT-effectiveness evaluation**

**SPONSOR: KNCV Tuberculosis Foundation**

**PRINCIPAL INVESTIGATOR: Katherine Fielding**

**INSTITUTION: London School of Hygiene & Tropical Medicine**

**COUNTRY PRINCIPAL Taye Letta**

**INVESTIGATOR:**

**INSTITUTION: National TB Programme in Ethiopia**

**DAYTIME HOURS TELEPHONE NUMBER(S): +251-911-367284**

**To the potential participant:** This consent form may contain words that you do not understand. Please ask the study staff to explain any words or information that you do not clearly understand. You may take home an unsigned copy of this consent form to think about or discuss with family or friends before making your decision.


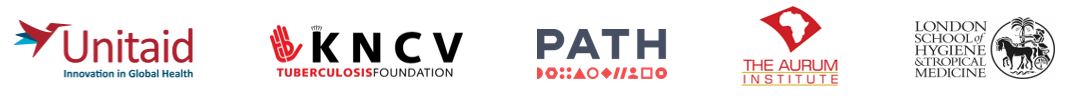


Hi, my name is ……………………………….. (Name of staff taking consent) and work as a …………………….. (Job title) at ……………………………….. (Health facility).

We would like to invite you to take part in a research study entitled “*ASCENT effectiveness evaluatio*n”.

1. Before agreeing to take part in this study, it is important that you read and understand (or have read to you) what is expected of you if you take part.
2. If you have any questions do not hesitate to ask me.
3. You should not agree to take part unless you are happy with all the things that may be asked of you.
4. If you agree to take part in this study, you will be asked to sign or thumbprint this document to confirm that you understand the study. You will be given a copy to keep.

**Why are we doing the study?**

- Research is a way to learn the answer to a question and use the answers to improve health care services.
- “TB” is a shorter way of saying tuberculosis, which is spread through coughing/ sneezing the TB germs.
- People with TB have to take treatment every day for 6 months and this can be very hard to do.
- Some people with TB have difficulties remembering to take their TB treatment everyday on time.
- This research study is part of a larger project called ASCENT and will be is looking at ways digital adherence technologies may help people take their TB treatment.
- Some people with TB in this study will be given “digital adherence technologies” which are designed to help remind people with TB to take their TB treatment daily and on time.
- You will be given one of these technologies to support you in your TB treatment. Every time you use the digital adherence technology to take your treatment, your doctor or health care provider will receive an automatic message that you took your daily TB pills.
- Sending this information about your TB pill-taking to your health care worker can help them to determine the best treatment approach for you and provide you with support if you need it.
- Even if you are using the digital adherence technology, you will still receive care from your health care worker.
- This study will also look at patients being cared for in the usual way by clinic health care workers.

There are three different parts (groups) to this study: smart pill box INTERVENTION, medication sleeve/label INTERVENTION and STANDARD OF CARE:

- Smart pill box INTERVENTION group: people are given a smart pill box (called evriMED) when they start TB treatment.
- Medication sleeve/label INTERVENTION group: people are given their treatment in customized packaging such as special sleeves or labels with a code to register your dose.
- The STANDARD OF CARE group: this means the usual standard of care is used to help patients like yourself to take TB treatment.

Your clinic is in the **medication sleeve/label INTERVENTION group.**

As you do not have a mobile phone and/or do not feel comfortable using a shared phone you are being offered **smart pill box** (sometimes called evriMED).

When you receive your medication at the health facility it will be put inside the pill box. The smart pill box is provided to you for free, but you will need to return it at the end of your treatment for TB. Every time you open the smart pill box, the electronic device inside the box sends a small signal (similar to an SMS or text) to your health care worker’s computer to record that you have opened the box to take your pills. Sending information about your pill-taking to your health care provider will help them to determine the best treatment approach for you.

The smart pill box will remind you (with a flashing green light or beeping sound) to take your medication daily and on time and not to miss any doses. There is also information on the box to help remind you of how to take your treatment. If you forget to take a dose your health care worker may either call, send you a message or visit you and offer help if you are having problems taking treatment.

The smart pill box only sends information about when the box is opened, how many times it is opened, and what the battery level of the device is. It is not able to not record or send any information about your family or your home. The smart pill box has a battery that lasts for several months and can be charged using the charging cable or at the clinic if needed.

**Why are we asking you to help?**

We are asking you to help in this research since you are to be treated for TB at this facility that is using this technology to assist patients like yourself to take TB treatment.

**How long do you have to be part of the study?**

- The study will take place in 78 clinics in 2 districts in Ethiopia
- The participants will include approximately 4000 adults.
- The total amount of time required for your participation in this study will be maximum of 12 months.

**What will happen if you take part in this study?**

Please remember that your participation is voluntary and you:

1. May refuse to participate in the study
2. Have the right to stop taking part in the study at any time.

If you agree to take part in this study, this is what will happen:

- We will ask you some questions about yourself (for example, age, address, your education). This talk may last about 5 minutes. You do not have to answer questions if you do not want to.
- Your health care provider will show you how to use the smart pill box.
- We will provide you with the box and a booklet that explains the use of the smart pill box
- The health care worker will collect some information such as your name, phone number (if case you have access to a phone) and TB registration number that is written on your treatment card. This information will be stored on a password protected a computer or tablet.
- Every day that you use the technology to help take your medication, your health care provider will receive a message
- The smart pill box is provided to you for free for your use during treatment. You will not be asked to pay for anything. You will return the box to the health facility once you have completed your treatment.
- As part of the study, you may receive text messages to the phone number you register, such as reminders or motivational messages. If you share a phone, you should only register this number if the people you share a phone with are people you can confide in and who can support you.
- In around 12 months time a research assistant will contact you directly, using the phone number you give us, taking care to be sure we are talking to you before we ask questions about your health. They will ask you whether you have had to restart TB treatment and whether anyone has found out, by accident, you are on TB treatment. This talk may last about 5 minutes.
- Depending on the type of TB you have, you may also be asked to attend this clinic to give a spit (sputum) sample to check that the TB has not come back. We will reimburse you 85ETB for travel to the clinic.
- We request your permission to access your medical records and laboratory results from this clinic or any other clinic where you sought care.
- There is no cost to you for taking part in the study.
- You will **NOT** be paid to take part in this study

**Use of your information**

- To be able to understand how these digital technologies may help people like yourself take and complete their TB treatment, researchers will be able to see your pill-taking information - when you open the box and what sorts of help such as SMS, phone call and home visits you have received.
- This information will be used to write a report or article about how these digital technologies can be used to improve the care given to TB patients like you around the world.
- This information will be anonymized, meaning that the researchers will never see your name, your address, or any other personal information that identifies you or your family with your pill-taking information.
- At the end of the project, study data will be made available to other researchers worldwide for non-commercial use to improve medical knowledge and patient care. Your study data will only refer to a study number and will not include your name, phone, address or any other personal identifier. While we believe the risk of someone identifying you is very small, we will take every precaution to ensure that other data elements such as the name or location of the health facility that could be used to identify you, will be modified or removed from the study data.

**What are the risks of participating in the study?**

- Some people may feel anxious or uncomfortable using these new technologies. These digital adherence technologies may be more “visible” to others in your home or workplace and may result in someone finding out that you are on TB treatment, by accident.
- You may reduce the chance of others guessing that you may have TB by keeping the **smart pill box** in a bag as you travel and at home.
- Some people may feel anxious or uncomfortable answering some questions, but you may skip any question that you are not comfortable with and you may stop the interview at any time.
- Friends and family members may react by becoming worried that you joined the study without consulting them.

**What are the benefits of participating in the study?**

- These digital adherence technologies may help you as patient to take your daily medication at a time and place that suits you best.
- A possible benefit from this new approach could mean that the frequency of your visits to the health facility will be lower than that for other TB patients for whom this is the only option.
- The technologies provide recent information on your pill-taking to the TB doctor or nurse to help them determine the most appropriate treatment approach for you. This way they can use this information to work with you to help make sure that you complete your treatment as intended to cure you of TB.
- Lastly, the information you provide and experience you have from using these digital technologies will be used to help improve the success of treatment for TB in patients like yourself.

**What happens if you do not agree to take part in this study?**

- If you do not want to be part of this study, simply say so.
- If at any time after signing up to take part in the study, you decide that you want to stop using the smart pill box you may do so. We would like to still access your medication records and speak to you 12 months after starting TB treatment – we will ask if this is ok with you.
- If at any time, you also decide to stop sharing your information, you may do so. We will delete your data.
- Stopping the study will not affect the kind of treatment you receive. You will continue to receive TB treatment by the health care workers at this health facility.

**How is the information collected during this study going to be kept confidential?**

- All information about you and your treatment collected during this study will be kept secure and confidential (kept secret). The data on the computer is kept on a secure server, encrypted and password protected. For research staff, your personal information (such as your name) is kept separate from other information you provide, in a password protected database. Actual responses to questions will only be identified using a study number that only the study staff can trace back to you.
- Reports about the study may be made to the government or the funder, but you will not be personally identified in any report about this study. In addition, we may be asked to provide data to the funder but your personal identifying information will not be included in that data.
- Any data reported in scientific journals will not include any information that identifies you as a participant in this study.
- The information might also be inspected by the Public Emergency and Health Research Directorate Institutional Review Boards of the Addis Ababa City Administration Health Bureau and Oromia Regional Health Bureau Public Emergency and Health Research Directorate Institutional Review Board who have your safety and privacy in mind.

**Study discontinuation**

If at any time the study is stopped by the funder, the Trial Advisory Group, or any of the relevant governing bodies, you will continue to receive treatment at this health facility according to the standard of care.

**Ethical approval**

This study protocol has been submitted to the Public Emergency and Health Research Directorate Institutional Review Boards of the Addis Ababa City Administration Health Bureau and Oromia Regional Health Bureau Public Emergency and Health Research Directorate Institutional Review Board and written approval has been granted by that committee. The study has been structured in accordance with the **Declaration of Helsinki** (last updated: October 2013), which deals with the recommendations guiding doctors in biomedical research involving human participants. A copy may be obtained from me should you wish to review it. The study is paid for by Unitaid.

If you have any further questions or queries about this project, please do not hesitate to contact Dr Gedion Tefera (ASCENT Country Project manager) on +251-0116630146 or Katherine Fielding, London School of Hygiene & Tropical Medicine, UK. (e-mail: [Katherine.fielding@lshtm.ac.uk](mailto:Katherine.fielding@lshtm.ac.uk)) OR you may also telephone Dr Amare W. Tadesse (ASCENT country Trial Manager) on +251-0116630164 during office hours.

**PARTICIPANT QUESTIONS?:**

Did the participant raise any questions?

YES/NO

If YES- What where they:

**(This information sheet will be available in local languages - Amharic and Afaan Oromootiin)**

I, hereby confirm that I have been informed by ………………..………..about the nature, conduct, benefits and risks of the study (*ASCENT-effectiveness evaluation*).

- I have received, read and understood the above written information (participant information sheet and informed consent) regarding the study.
- I am aware that the results of the study, including personal details regarding my sex, age, date of birth, initials and diagnosis will be anonymously processed into a study report.
- I may, at any stage, without prejudice, withdraw my consent and participation in the study.
- I have had sufficient opportunity to ask questions and (of my own free will) declare myself prepared to participate in the study.

**PARTICIPANT**

________________________________ ___________________________________

Participant’s Name (print) Participant’s Signature

Date: __________________________

I,………………………………(insert name of research staff), herewith confirm that the above participant has been fully informed about the nature, conduct and risks of the above study.

**HEALTH CARE WORKER**

____________________________ ___________________________

Study Staff Member (print) Study Staff Member’s Signature

Date: _____________________________

**WITNESS* (If applicable):**

______________________ ___________________________

Print Name Signature:

Date: _____________________________

Contact details:

Trial Manager: Dr Amare W Tadesse +251-0116630164

Country Investigator: Taye Letta +251-911367284.

**PARTICIPANT Information Sheet and CONSENT Form for ETHIOPIA
Effectiveness evalUation – STANDARD OF CARE**


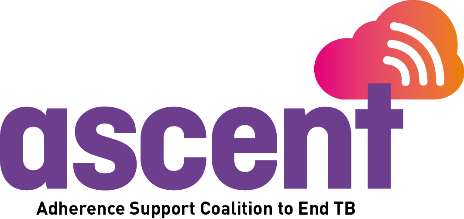


**STUDY TITLE: Adherence Support Coalition to End TB: evaluation of digital adherence technologies for TB treatment in Ethiopia**

**SHORT TITLE: ASCENT-effectiveness evaluation**

**SPONSOR: KNCV Tuberculosis Foundation**

**PRINCIPAL INVESTIGATOR: Katherine Fielding**

**INSTITUTION: London School of Hygiene & Tropical Medicine**

**COUNTRY PRINCIPAL Taye Letta**

**INVESTIGATOR:**

**INSTITUTION: National TB Programme in Ethiopia**

**DAYTIME HOURS TELEPHONE NUMBER(S): +251-911-367284**

**To the potential participant:** This consent form may contain words that you do not understand. Please ask the study staff to explain any words or information that you do not clearly understand. You may take home an unsigned copy of this consent form to think about or discuss with family or friends before making your decision.


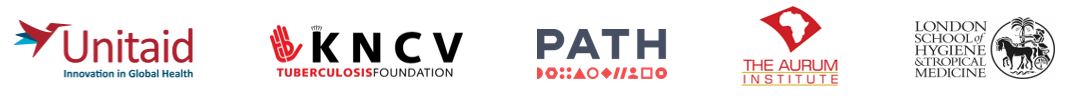


Hi, my name is ……………………………….. (Name of staff taking consent) and work as a …………………….. (Job title) at ……………………………….. (Health facility).

We would like to invite you to take part in a research study entitled “*ASCENT effectiveness evaluatio*n”.

1. Before agreeing to take part in this study, it is important that you read and understand (or have read to you) what is expected of you if you take part.
2. If you have any questions do not hesitate to ask me.
3. You should not agree to take part unless you are happy with all the things that may be asked of you.
4. If you agree to take part in this study, you will be asked to sign/thumbprint this document to confirm that you understand the study. You will be given a copy to keep.

**Why are we doing the study?**

- Research is a way to learn the answer to a question and use the answers to improve health care services.
- “TB” is a shorter way of saying tuberculosis, which is spread through coughing/ sneezing the TB germs.
- People with TB have to take treatment every day for 6 months and this can be very hard to do.
- Some people with TB have difficulties remembering to take their TB treatment every day on time.
- This research study is part of a larger project called ASCENT and will be looking at ways digital adherence technologies may help people take their TB treatment.
- Some people with TB in this study will be “digital adherence technologies” and are designed to help remind people to take their TB treatment daily and on time.
- This study will also look patients who are being cared for in the usual way by clinic health care workers.

There are three different parts (groups) to this study: smart pill box INTERVENTION, medication /label INTERVENTION and STANDARD OF CARE.

The STANDARD OF CARE group: this means the usual standard of care is used to help patients like yourself to take TB treatment.

Smart pill box INTERVENTION group: people are given a smart pill box (called evriMED) when they start TB treatment.

Medication label INTERVENTION group: people are given their treatment in customized packaging such as special s or labels with a code to register your dose.

Your clinic is in the **STANDARD OF CARE group**

You are in the STANDARD OF CARE group. This means the usual standard of care is used to assist patients like yourself to take TB treatment. This will include a health care worker observing you take treatment at the health facility, or a treatment supporter observing you take treatment.

**Why are we asking you to help?**

We are asking you to help in this research since you are to be treated for TB at this facility that is using standard of care to assist patients like yourself to take their TB treatment.

**How long do you have to be part of the study?**

- The study will take place in 78 clinics in 2 districts in Ethiopia
- The participants will include approximately 4000 adults.
- The total amount of time required for your participation in this study will be maximum of 12 months.

**What will happen if you take part in this study?**

Please remember that your participation is voluntary and you:

1. May refuse to participate in the study
2. Have the right to stop taking part in the study at any time.

If you agree to take part in this study, this is what will happen:

- e will ask you some questions about yourself (for example, age, address, your education). This talk may last about 5 minutes. You do not have to answer questions if you do not want to.
- You will then receive your TB care as is standard practice in this facility.
- In around 12 months time a research assistant will contact you directly, using the phone number you give us, taking care to be sure we are talking to you before we ask questions about your health. They will ask you whether you have had to restart TB treatment and whether anyone has found out, by accident, you are on TB treatment. This talk may last about 5 minutes.
- Depending on the type of TB you have, you may also be asked to attend this clinic to give a spit (sputum) sample to check that the TB has not come back. We will reimburse you 85ETB for travel to the clinic.
- We request your permission to access your medical records and laboratory results from this clinic or any other clinic where you sought care.
- There is no cost to you for taking part in the study.
- You will **NOT** be paid to take part in this study

**What are the risks of participating in the study?**

- Some people may feel anxious or uncomfortable answering some questions, but you may skip any question that you are not comfortable with and you may stop the interview at any time.
- Friends and family members may react by becoming worried that you joined the study without consulting them.

**What are the benefits of participating in the study?**

There may be no direct benefits to you when taking part in this study. The information you provide will be used to help improve the success of treatment for TB in patients like yourself.

**What happens if you do not agree to take part in this study?**

- If you do not want to be part of this study, simply say so.
- You may withdraw (stop taking part) from the study at any time. If you withdraw from the study, we will not contact you by phone in 12 months time. We would like to still access your medication records and will ask if this is ok with you.
- Stopping the study will not affect the kind of treatment you receive. You will still continue to receive TB treatment by the health workers at this health facility.

**How is the information collected during this study going to be kept confidential?**

- All information collected about you and your treatment during this study will be kept secure and confidential (kept secret). The data on the computer is kept on a secure server, encrypted and password protected. For research staff, your personal information (such as your name) is only available to study staff and kept separate from other information you provide in a locked and secured cabinet and a password protected database. Actual responses to questions will only be identified using a study number that only the study staff can trace back to you.
- Reports about the study may be made to the government or the funder, but you will not be personally identified in any report about this study.
- In addition, we may be asked to provide data to the funder but your personal identifying information will not be included in that data.
- Any data reported in scientific journals will not include any information that identifies you as a participant in this study.
- At the end of the project, study data will be made available to other researchers worldwide for non-commercial use to improve medical knowledge and patient care. Your study data will only refer to a study number and will not include your name, phone, address or any other personal identifier. While we believe the risk of someone identifying you is very small, we will take every precaution to ensure that other data elements such as the name or location of the health facility that could be used to identify you, will be modified or removed from the study data.
- The information might also be inspected by the Public Emergency and Health Research Directorate Institutional Review Boards of the Addis Ababa City Administration Health Bureau and Oromia Regional Health Bureau Public Emergency and Health Research Directorate Institutional Review Board who have your safety and privacy in mind.

If you are willing to participate you will have to sign the consent form below. You can ask us questions about anything before deciding to participate or not.

**Study discontinuation**

If at any time the study is stopped by the funder, the Trial Advisory Group, or any of the relevant governing bodies, you will continue to receive treatment at this health facility according to the standard of care.

**Ethical approval**

This study protocol has been submitted to the Public Emergency and Health Research Directorate Institutional Review Boards of the Addis Ababa City Administration Health Bureau and Oromia Regional Health Bureau Public Emergency and Health Research Directorate Institutional Review Board and written approval has been granted by that committee. The study has been structured in accordance with the **Declaration of Helsinki** (last updated: October 2013), which deals with the recommendations guiding doctors in biomedical research involving human participants. A copy may be obtained from me should you wish to review it. The study is paid for by Unitaid.

If you have any further questions or queries about this project, please do not hesitate to contact Dr Gedion Tefera (ASCENT Country Project manager) on +251-0116630146 or Katherine Fielding, London School of Hygiene & Tropical Medicine, UK. (e-mail: [Katherine.fielding@lshtm.ac.uk](mailto:Katherine.fielding@lshtm.ac.uk)) OR you may also telephone Dr Amare W. Tadesse (ASCENT country Trial Manager) on +251-0116630164 during office hours.

**PARTICIPANT QUESTIONS?:**

Did the participant raise any questions? YES/NO

If YES- What where they:

**(This information sheet will be available in local languages - Amharic and Afaan Oromootiin)**

- I, hereby confirm that I have been informed by ………………..………..about the nature, conduct, benefits and risks of the study (*ASCENT-effectiveness evaluation*).
- I have received, read and understood the above written information (participant Information leaflet and Informed consent) regarding the study.
- I am aware that the results of the study, including personal details regarding my sex, age, date of birth, initials and diagnosis will be anonymously processed into a study report.
- I may, at any stage, without prejudice, withdraw my consent and participation in the study.
- I have had sufficient opportunity to ask questions and (of my own free will) declare myself prepared to participate in the study.

**PARTICIPANT**

________________________________ ___________________________________

Participant’s Name (print) Participant’s Signature

Date: __________________________

I,………………………………(insert name of research staff), herewith confirm that the above participant has been fully informed about the nature, conduct and risks of the above study.

**HEALTH CARE WORKER**

____________________________ ___________________________

Study Staff Member (print) Study Staff Member’s Signature

Date: _____________________________

**WITNESS* (If applicable):**

______________________ ___________________________

Print Name Signature:

Date: _____________________________

Contact details:

Trial Manager: Dr Amare W Tadesse +251-0116630164

Country Investigator: Taye Letta +251-911367284.
